# An *ex vivo* human placental explant model for controlled exposure studies

**DOI:** 10.1016/j.xpro.2025.104055

**Published:** 2025-08-29

**Authors:** Nida Ozarslan, Lin Li, Sirirak Buarpung, Joshua F. Robinson, Stephanie L. Gaw

**Affiliations:** 1 Division of Maternal-Fetal Medicine, Department of Obstetrics, Gynecology & Reproductive Sciences, University of California, San Francisco, San Francisco, CA 94158, USA; 2 Center for Reproductive Sciences, Department of Obstetrics, Gynecology, and Reproductive Sciences, University of California, San Francisco, San Francisco, CA 94143, USA

**Keywords:** Cell Biology, Developmental biology, Immunology

## Abstract

The human placental explant model preserves the placenta’s natural structure, enabling studies on whole-tissue response to exposures or growth conditions. It outlines procedures for placental collection, isolation, and culturing, followed by exposure to conditions of interest. Additionally, it details sample collection of explant tissue and culture supernatant after exposure for downstream assessments of placental function. This protocol provides a framework for evaluating *ex vivo* responses of human placental tissue.

For complete details on the use and execution of this protocol, please refer to Gonzalez et al.^1^

## Highlights

• Steps for establishing human placental explant models across gestation
• Instructions for maintaining the original structure of placental villous tissue
• Investigate the functional impact of experimental exposures in placental tissue

## Abstract (editor highlights)

Publisher’s note: Undertaking any experimental protocol requires adherence to local institutional guidelines for laboratory safety and ethics.

## Graphical abstract

**Figure:**
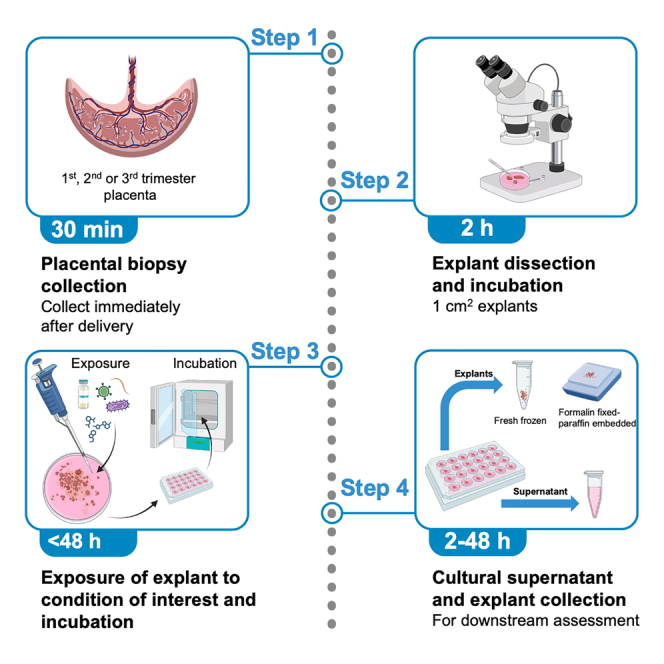

## Before you begin

The protocol below describes specific steps for isolating human placental explants from placental biopsies after delivery and setting up the explants for experimental exposures and conditions of interest.

Safety data of multiple drugs and vaccines are limited in pregnancy as pregnant individuals are mostly excluded from randomized clinical trials. It is challenging to study the direct impact of drugs, chemicals, infectious agents, or other exposures on the human placenta, a unique maternal-fetal interface that is crucial for fetal development and healthy pregnancy. While no in vitro or ex vivo model can perfectly replicate the complex physiology of the maternal-fetal unit during pregnancy, the explant model maintains the physiological condition and structure of the placenta, serving as a useful method to investigate placental responses to experimental conditions.

This protocol can be used to test the impact on placental tissue function of various factors under controlled conditions, including chemicals, hormones, drugs, nutrients, toxins, pathogens, and other conditions of interest.

### Innovation

The placental explant model has been used for many decades.^2^ In this protocol, we aimed to describe a comprehensive, step-by-step guide to harvest placental explants across gestation.

### Institutional permissions

Human placental biopsies were obtained from elective terminations of pregnancy (1^st^ and 2^nd^ trimester) or uncomplicated pregnancies (3^rd^ trimester) that delivered at the University of California, San Francisco (UCSF) Medical Center. The study was approved by the institutional review board of the UCSF, and written consent was obtained from all participants. Ensure you have received the appropriate institutional permission to work with human samples before proceeding with this protocol.

**Figure 1:**
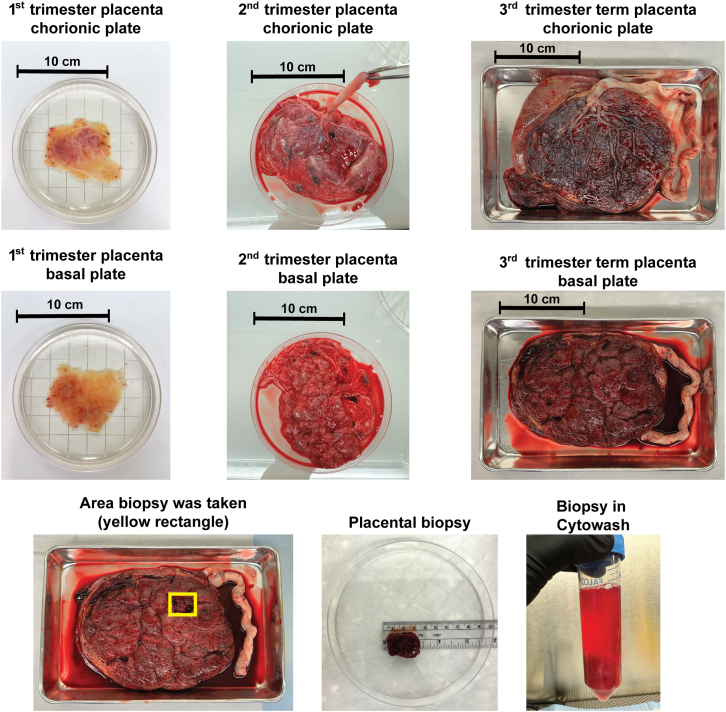
Gross placental anatomy of 1^st^, 2^nd^, and 3^rd^ trimester placentas and collection of placental biopsies 1^st^ (11 week) and 2^nd^ trimester (20 weeks) samples inside a 15 cm diameter Petri dish. 3^rd^ trimester (37 weeks) term placenta inside a 28 cm × 18 cm metal tray. In 2^nd^ and 3^rd^ trimester placentas, the chorionic plate houses the cord insertion, whereas the basal plate has a more lobulated appearance, this differentiation might not be visualized in 1^st^ trimester placenta. Obtain placental biopsies from areas free of visible infarction, calcification, hematoma, or tears if possible. Place biopsy immediately in cold Cytowash and store on ice. The scale bars represent 10 cm.

### Preparation of reagents and solutions

**Timing: 1 h**

1. Preparation of Cytowash and serum-free medium.
  a. Following the material tables, prepare 500 mL of Cytowash stock solution and 200 mL of serum-free medium stock solution.
  b. Filter each through separate 0.22 μm pore size vacuum filtration systems.
  c. Store the filtered Cytowash and serum-free medium stock solution at 4°C.
2. Preparation of explant culture medium.
  a. Prepare explant culture medium (serum-free medium with 10% FBS) by adding 5 mL of FBS to 45 mL of serum-free medium.
  b. Mix thoroughly with a serological pipette.
3. Preparation of exposure of interest solution.
  a. Prepare stock solution of exposure of interest such as drug, chemical, hormone, nutrient or pathogen, if applicable.

***Optional:*** The exposure of interest will be customized for individual studies.

### Collection of placental biopsies

**Timing: 0.5 h**

4. Collect placenta from elective termination of pregnancy (5–24 weeks) or from normal births immediately after delivery.

**CRITICAL:** Obtain placental biopsies within one hour of delivery, ideally immediately after collection. The optimal window to collect placental biopsies has been described as 10 min to avoid any changes that might occur due to hypoxia.^3^ A delay in this step can influence tissue viability and thereby impact experimental outcomes.

**CRITICAL:** Particularly for 3^rd^ trimester placenta, we prefer late preterm or early term deliveries (34 weeks 0 days to 37 weeks 0 days) by cesarean section due to the physiological increase in senescence markers with advancing gestational age.^4^

5. Obtain placental biopsies measuring a maximum of 3 cm × 3 cm × 3 cm using surgical scissors.

**CRITICAL:** For 3^rd^ trimester placentas, using the umbilical cord insertion as the center and the start of the amniochorionic membrane as the outer boundary, biopsies should be collected from the mid-zone of the placenta, selecting cotyledons that are free of visible infarction, calcification, hematoma, or tears (Figure 1).

***Note:*** Considering the smaller size of the 1^st^ and 2^nd^ trimester placentas, the collection of 3 cm × 3 cm × 3 cm biopsies might not be feasible; instead, try to obtain smaller but as many biopsies as necessary to generate sufficient explants for your experimental plan.

6. Briefly rinse with at least 500 mL cold DPBS to remove excess blood.

***Alternatives:*** The placental biopsies can be rinsed using cold PBS if DPBS is not available.

7. Place biopsies in a 50 mL conical, and fill up to 40 mL line with cold Cytowash (Figure 1).

**CRITICAL:** Keep the biopsies in Cytowash and store them on ice prior to proceeding with explant dissection.

### General laboratory preparation

**Timing: variable**

8. Prewarm explant culture medium at 37°C.
9. Sterilize fine scissors and forceps and place them on ice before use.
10. Set up the dissection microscope.

## Key resources table

**Table:** 

REAGENT or RESOURCE	SOURCE	IDENTIFIER
**Biological samples**		

Human placental tissue	UCSF	N/A

**Chemicals, peptides, and recombinant proteins**		

Fetal bovine serum	Sigma-Aldrich	Cat#F0926
Penicillin-streptomycin (10,000 U/mL)	Gibco	Cat#15140122
Gentamicin (50 mg/mL)	Gibco	Cat#15750060
DPBS	Gibco	Cat#14190250
DMEM, high glucose	Gibco	Cat#11965092
Glutamine plus (200 mM)	Atlanta Biologicals	Cat#B90210
Nutridoma (100×)	Roche	Cat#11011375001
Sodium pyruvate (100 mM)	Gibco	Cat#11360070
HEPES buffer (1 M)	Gibco	Cat#15630080
SYBRTM Green qPCR Master Mix	Applied Biosystems	Cat#43-676-59
10% Neutral Buffered Formalin	RPI	Cat#F10800–500.0
Ethanol 200PRF	Decon Labs	Cat#V1001

**Software and algorithms**		

LAS Science Microscope Software	Leica	https://www.leica-microsystems.com

**Other**		

0.22 μm pore size, sterile vacuum filtration system, 500 mL bottle top	–	–
50 mL conical tube	–	–
1.5 mL microcentrifuge tube	–	–
Petri dish (150, 100, and 35 mm)	–	–
24-well plate	–	–
Serological pipette (5, 10, and 25 mL)	–	–
Measuring cylinders	–	–
Pipette gun	–	–
Single-channel pipette and pipette tips (10, 200, and 1,000 μL)	–	–
Surgical scissors	–	–
Micro scissors	–	–
Forceps	–	–
Ruler	–	–
Water bath	–	–
Ice bucket	–	–
Sterile CO_2_ incubator	–	–
4°C Fridge	–	–
Dissection microscope	–	–

## Materials and equipment

**Table:** Cytowash

Reagent	Final concentration	Amount
DMEM, high glucose	95.4%	477 mL
Fetal bovine serum	2.5%	12.5 mL
Glutamine plus (200 mM)	1%	5 mL
Penicillin-streptomycin (10,000 U/mL)	1%	5 mL
Gentamicin (50 mg/mL)	0.1%	0.5 mL
**Total**	**100%**	**500 mL**

Cytowash can be stored at 4°C for up to one month.

**Table:** Serum-free medium

Reagent	Final concentration	Amount
DMEM, high glucose	94.75%	189.5 mL
Glutamine plus (200 mM)	1%	2 mL
Sodium Pyruvate (100 mM)	1%	2 mL
HEPES Buffer (1 M)	1%	2 mL
Gentamicin (50 mg/mL)	0.25%	0.5 mL
Nutridoma (100×)	2%	4 mL
**Total**	**100%**	**200 mL**

Serum-free medium can be stored at 4°C for up to one month.

## Step-by-step method details

This part of the protocol describes the detailed dissection and culture of placental explants from the human placental biopsies. It is followed by the procedure for exposure of placental explants to the condition of interest. After treatment, the placental explants and culture supernatant can be collected for further downstream assessment.

### Explant dissection and incubation

**Timing: 2 h**

This section describes methods to isolate the placental villi explants from the placental biopsies.

1. Prepare culture plates.
  a. Determine the number and position of the wells needed in a 24-well plate.
  b. Plan the plate layout.
  c. Add 500 μL prewarmed explant culture medium into designated wells.

***Note****:* If multiple time points are involved, use an individual plate for each time point (e.g., 30 min, 4 h, etc.).

**Figure 2:**
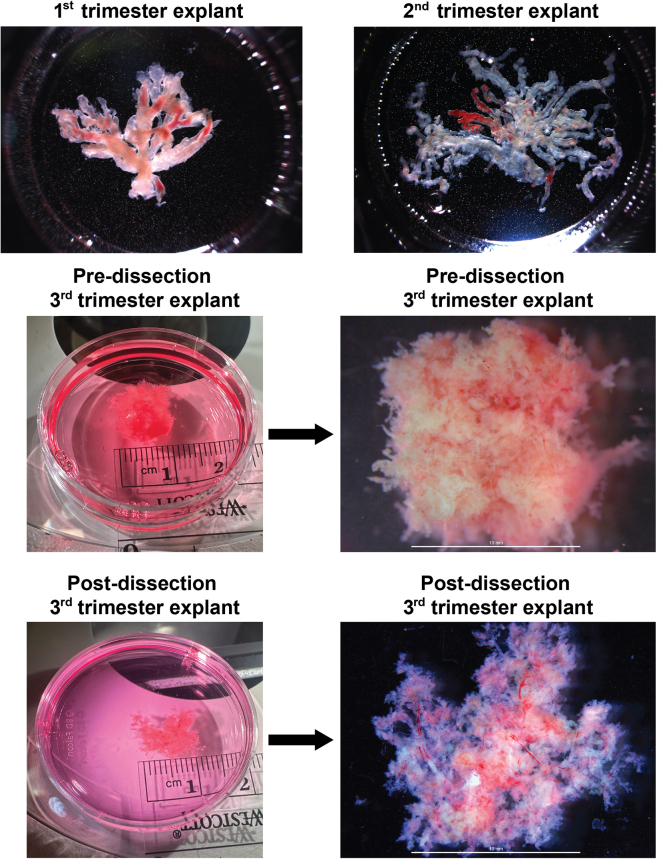
Placental explants After cutting 1 cm × 1 cm pieces from placental biopsies, dissect chorionic villi carefully under the dissecting microscope to reduce the density of villi and allow media access to all surfaces. The explants should have freely floating, spread out villi after dissection. The scale bars represent 1 cm.

2. Take out the placental biopsy from 50 mL conical and place on 100 mm petri dish on ice with cold Cytowash.
  a. Remove any basal plate if available.
3. Place the remaining biopsy under a dissection microscope and identify stem villi.
4. Obtain smaller 1 cm^2^ pieces (Figure 2).
5. Wash the smaller 1 cm^2^ pieces with ice cold Cytowash 2–3 times to rinse out any remaining blood.
6. Transfer the small biopsy piece into a 35 mm petri dish with ice cold Cytowash.
7. Dissect the chorionic villi carefully starting from the stem villi, allowing the villi to spread out (Figure 2).

***Note:*** The villi are extremely fragile and should be treated gently.

**CRITICAL:** The villi should not be excessively dense. Carefully dissect the villi and allow them to spread out so that the culture media can reach all surfaces equally.

**CRITICAL:** During the dissection, first identify the stem villi and gently dissect following the branches originating from that stem villi to prevent damage to the branching villi. Investigate the villi under high magnification using a dissection microscope and confirm the grossly intact structure before proceeding to the next steps.

8. After dissection is complete, trim the villi to 10 mm × 10 mm if necessary.
9. Put each piece of villi in a well of a 24-well plate containing 500 μL prewarmed explant culture medium and spread the villi out.
10. Place culture plates in a standard sterile CO_2_ incubator (5% CO_2_) at 37°C for 30 min before exposure.

### Explant exposure

**Timing: <48 h**

These steps describe the treatment of placental explants with tested substances of interest.

11. Take out the 24-well plate from the incubator.
12. Remove 200 μL medium from each well, leaving 300 μL in each well to maintain semi-conditioned media for optimal explant viability.

**CRITICAL:** When removing or adding the medium, avoid disturbing the placental explants and manipulate as little as possible.

13. Add 200 μL of the prepared exposure of interest solution at the desired concentration or 200 μL medium as a control to each well.
14. Gently shake the plate to mix the medium.
15. Place the plates back in the standard sterile CO_2_ incubator (5% CO_2_) at 37°C or the respective incubation conditions of interest for up to 48 h depending on your experimental design.

***Optional:*** Human placental explants can be incubated for longer than 48 h; however, their viability will significantly decrease with time.

### Cultural supernatant and explant collection

**Timing: 2–48 h**

This section describes the collection of the experimental samples for downstream assessment. Placental explants can be collected by obtaining fresh frozen and/or formalin fixed-paraffin embedded samples, for conducting RT-PCR, RNA-seq, RNA FISH, or IHC. Additionally, culture supernatant can be collected for performing viability, cytotoxicity, or cytokine measurement assays, and more.

16. Remove placental explants from 24 well plate.
17. Rinse briefly with DPBS.
18. Either place directly in a 1.5 mL microcentrifuge tube and store at −80°C or fix in 10% neutral buffered formalin for 24 h at 20°C to 24°C, wash in 70% ethanol, and store at 4°C until undergoing further processing for paraffin embedding.

***Optional:*** Explant samples and culture supernatant can be collected and processed with different methods depending on planned downstream assessments.

19. Obtain 400 μL of culture supernatant from each well and transfer to a 1.5 mL microcentrifuge tube.
  a. Store the culture supernatant at −80°C for downstream assessment.

## Expected outcomes

This protocol is useful for isolating human placental explants from 1^st^, 2^nd,^ and 3^rd^ trimester placenta resembling images in Figure 2. Placental explants can be exposed to various factors, including chemicals, hormones, drugs, nutrients, toxins, pathogens, and other substances which enables the investigation of the direct impact on the human placenta under controlled conditions while preserving its unique structure.

## Limitations

The protocol works best with freshly obtained placenta, ideally within 15 min of delivery up to 1 h. Delays in collection and processing will affect the viability of the explants and downstream assays. For 3^rd^ trimester placenta, non-labored, cesarean section deliveries should be preferred over labored or vaginally delivered placenta. We also prefer late preterm or early term (34 weeks 0 days to 37 weeks 0 days) placentas to optimize the viability of the tissue. Placental explants incubated for over 48 h should be carefully monitored for morphological changes since some destruction might start to occur. Inevitable biological differences across explants obtained from different biopsies should be carefully taken into consideration when interpreting results.

## Troubleshooting

### Problem 1

At the beginning, obtaining explants with stem and branching villi can be challenging, particularly in 3^rd^ trimester placenta due to its dense structure.

### Potential solution

Start practicing dissection with smaller pieces of placental explants rather than 1 cm^2^ explants. Instead of cutting any villi, try gently distancing the villi from each other beginning from the stem villi by slowly moving the micro scissors through the villi.

### Problem 2

The clinical site where placental biopsies are donated and the research lab are far from each other.

### Potential solution

Place the placental biopsy immediately in cold cytowash and transfer it within an insulated transfer bag with frozen ice packs to help maintain temperature. The protocol would ideally be started within 1 h.

### Problem 3

Inter-individual biological differences can introduce variability in responses.

### Potential solution

Include technical replicates for each placenta and biological replicates from multiple placentas. Our group typically conducts experiments with at least 3 technical replicates from each placenta, and at least 4 placentas for each experiment. Normalize downstream data and apply appropriate statistical models to account for variability.

### Problem 4

Moving explants while pipetting can alter positioning or cause structural damage.

### Potential solution

Carefully pipette from the side of the well to remove or add media without touching the explant.

### Problem 5

Inadequate sterility can introduce microbial contamination, especially during dissection and culture setup.

### Potential solution

Perform all procedures in a certified biosafety cabinet using sterile instruments, reagents, and media. The initial wash of the placental biopsy with DPBS is critical to remove the majority of contaminating microbes. Ensure that the volume is at least 500 mL with agitation to maximize the dilution and removal of contaminants. Regularly monitor cultures and facilities for contamination.

## Resource availability

### Lead contact

Further information and requests for resources and reagents should be directed to and will be fulfilled by the lead contact, Stephanie L. Gaw (stephanie.gaw@ucsf.edu).

### Technical contact

Technical questions on executing this protocol should be directed to and will be answered by the technical contact, Nida Ozarslan (nida.ozarslan@ucsf.edu).

### Materials availability

This study did not generate new unique reagents.

### Data and code availability

This study did not generate or analyze any datasets or code.

## Acknowledgments

We thank Raha Sadeghi for the collection of the 1^st^ and 2^nd^ trimester placenta samples. This work was funded by the 10.13039/100000002National Institutes of Health (K08AI141728 and R01HD111582).

## Author contributions

N.O., L.L., and S.B. conducted the experiments and wrote the manuscript. J.F.R. and S.L.G. obtained funding and supervised the work. All authors revised the manuscript.

## Declaration of interests

The authors declare no competing interests.
